# Nonrestorative Management of Dental Caries

**DOI:** 10.3390/dj9100121

**Published:** 2021-10-18

**Authors:** Ollie Yiru Yu, Walter Yu-Hang Lam, Amy Wai-Yee Wong, Duangporn Duangthip, Chun-Hung Chu

**Affiliations:** Faculty of Dentistry, The University of Hong Kong, Hong Kong; retlaw@hku.hk (W.Y.-H.L.); drawong@hku.hk (A.W.-Y.W.); dduang@hku.hk (D.D.); chchu@hku.hk (C.-H.C.)

**Keywords:** dental caries, caries management, nonrestorative management, caries prevention

## Abstract

The World Dental Federation (FDI) policy statement in 2016 advocated evidence-based caries-control measures for managing dental caries. The caries management philosophy has shifted from the traditional surgical manners to minimal intervention dentistry. Minimal intervention dentistry aims to extend the longevity of natural teeth. It places the nonrestorative approaches as a priority. The nonrestorative approaches for caries management aim to tackle the etiological factors of dental caries. Caries can be prevented or reversed by restricting the sugar intake and its frequency in the diet, improving oral hygiene practices, and using fluoride toothpaste. This article aims to present strategies for the nonrestorative management of dental caries, which are divided into four components to address the different etiological factors of dental caries. The first component is controlling dental plaque. Strategies for plaque control include oral hygiene instruction, motivational interviewing, mechanical plaque control, and chemical plaque control. The second component for nonrestorative management is reducing the risk of caries by identifying caries risk factors and protective factors, assessing personal caries risk, and customizing a treatment plan. Evidence-based measures for caries prevention include using fluoride, and dental sealants should be provided. The third component includes topical treatment to remineralise early carious lesions. The last component is long-term follow-up. Appropriate strategy adoption for the nonrestorative management of dental caries prolongs the life span of the teeth and sustains the good oral health of patients.

## 1. Introduction

Dental caries remains prevalent worldwide. A survey in 2015 found 2.4 billion people have untreated caries, affecting 34% of adults and 8% of children [1]. Although dental health care providers have put much effort into and have implemented various strategies to control dental caries, the incidence of caries has remained similarly high over the past decades [2]. Only a 4% decrease was found in caries prevalence compared to thirty years ago [2].

Although the prevalence of caries seems to be slightly lower, the number tooth surfaces that are vulnerable to caries has increased [3]. Patients who have periodontal disease have a high chance of having both root caries and coronal caries [4]. The increased exposed root surfaces resulting due to periodontal disease may lead to a higher incidence of root caries [4]. The increased plaque retention due to the cavitated lesions, poor oral hygiene, or the co-occurrence of several risk behaviours may contribute to the increased incidence of coronal caries in periodontal patients [4]. Orthodontic treatment was believed to be associated with dental caries because orthodontic appliances facilitate the accumulation of the dental biofilm. Early caries lesions or white spot lesions are the most common complications of orthodontic treatment [5]. The reported incidence rates of white spot lesions ranges from 38% to 73% in orthodontic patients [6,7].

The ineffectiveness of caries management strategies may be attributed to the discrepancy between the previous understanding and the true characteristics of dental caries. In the past, dentists managed caries as an infectious disease caused by pathogenic microorganisms that can be transmitted from one person to another person [8]. Hence, the treatment objectives were to completely disinfect the infectious sites, kill the pathogens, remove potential breeding sites, and prevent the recurrence of infection. Caries treatment often focused on complete caries removal and cavity restoration right after detecting caries lesions. Removing healthy dental hard tissue for caries prevention was also recommended [9]. This treatment philosophy for dental caries is a fundamentally operative treatment that takes place in a surgical manner.

The World Dental Federation (FDI) policy statement in 2016 advocated evidence-based caries-control measures for caries management [10]. The surgical management of dental caries that tackles the symptoms and signs of the disease with dental restoration is no longer a common model for dental care. It does not address the disease’s etiological factors and thus caries prevention or control cannot be attained. The treatment outcome basically relies on the success of dental restorations, which have limited longevity. A study found that the average longevity of resin composite restoration was only 6 years [11]. A systematic review that included 1844 amalgam restoration and 1642 resin composite restoration found that the annual failure rates were 1.71% for amalgam restoration and 3.17% for resin composite [12]. Patients with a high caries risk had a significantly higher dental restoration failure compared to patients with a low caries risk [13]. These studies showed that the surgical management for caries was unsatisfactory in terms of controlling dental caries.

The contemporary philosophy of caries management has shifted from the traditional surgical approach to a novel concept that is more oriented toward controlling etiological factors due to the current understanding of dental caries as a non-communicable chronic behaviour—mediated biofilm disease [14]. The ecological plaque hypothesis has been the mainstream the explanation for the relationship between dental caries and the oral microbiota [15]. A healthy oral microbiota benefits the host by immunological initiation, the down-regulation of inflammatory responses, and the regulation of exogenous microorganism colonisation [16]. The changes to the local environment, such as increased sugar intake, shifts the composition of the oral microbiota to adapt to the new environment. The enrichment of the acidogenic and aciduric microorganisms break the homeostasis of the micro-community and cause dental caries [15]. The ecological plaque hypothesis indicates that caries can be controlled by improving oral hygiene, by reducing the targeting pathogenic microorganism and interfering with the environmental factors that are suitable for the cariogenic microorganism [15].

The current cariology philosophy is also in agreement with minimal intervention dentistry that aims to preserve dental structure and pulpal vitality and thus extend the lifespan of the teeth [14]. The ultimate goal is to allow the disease to heal with the improvement of oral health. It emphasizes the nonrestorative intervention to inhibit the mineral loss of carious lesions at all stages of caries development [17]. Nonrestorative management is therefore a priority in caries management. This article presents strategies for the nonrestorative management of dental caries. We classify t nonrestorative management strategies into four essential components by addressing the different etiological factors of dental caries, namely controlling dental plaque, reducing caries risk, preserving dental hard tissue, and engaging in long-term follow-up. These caries-control strategies along with the support of high-level evidence, such as systematic reviews of clinical trials and randomized clinical trials, were discussed under each component of nonrestorative management of dental caries. This article aims to provide an overview of the evidence-based nonrestorative management for dental caries.

## 2. The Nonrestorative Management of Dental Caries

**Figure 1:**
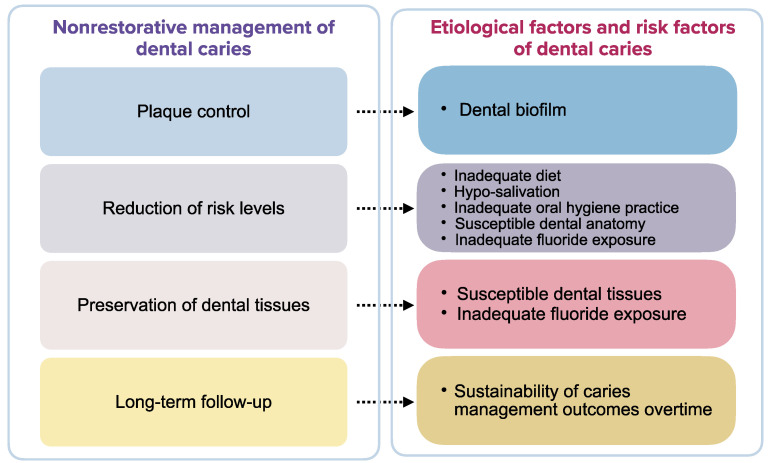
Essential components of nonrestorative management of dental caries.

Dental caries is a non-communicable disease [18]. The etiological factors that directly contribute to the progression of dental caries include the biofilm in the dental plaque, diet, susceptible dental hard tissue, and time [19]. The nonrestorative management of dental caries should address the etiological factors and risk factors of caries [20]. Therefore, we have categorized the essential components for the nonrestorative management of dental caries into dental plaque control, reducing caries risk, preserving dental hard tissue, and long-term maintenance (Figure 1).

### 2.1. Control of Dental Plaque

Oral hygiene education is the provision of oral health information. It refers to the advice or instructions given to improve people’s oral hygiene behaviour or their oral condition. The World Health Organization (WHO) set goals toward oral hygiene education to reduce the risk of oral disease and to promote oral health [21]. Traditional oral hygiene education was a common dental practice, but unfortunately, its effects on oral hygiene improvement and caries control are questionable [22]. In addition, no long-term results of the effectiveness of oral hygiene education on plaque control or caries prevention are available [22]. Motivational interviewing was proposed to improve the effectiveness of oral hygiene education [23]. This is a patient-centred counselling strategy to improve a patient’s intrinsic motivation and to change the patient’s oral health behaviour. A systematic review found that motivational interviewing was more effective in improving oral hygiene than traditional oral hygiene education [24]. A study found motivational interviewing reduced caries incidence by 29% in children [25]. A study reported motivational interviewing had inequivalent effects on patients with different levels of caries risk. Although motivational interviewing could effectively prevent caries in patients with high caries risk, its effect was insubstantial in patients with low caries risk [26].

Tooth brushing is the most common oral hygiene practice for mechanical plaque control. It is a fundamental behaviour for maintaining and promoting oral health [27]. The American Dental Association states that powered and manual toothbrushes are both effective at removing the dental plaque that causes dental caries [28]. A review concluded that powered toothbrushes are superior to manual toothbrushes for both short-term and long-term plaque control [29]. Tooth brushing behaviour is a factor associated with caries development. People with irregular toothbrushing habits had a higher incidence of caries than those with regular toothbrushing habits [30]. People who brushed their teeth less than twice a day had a higher caries incidence than those who brushed at least twice a day. Interestingly, the incidence of dental caries remained similar when the frequency of tooth brushing was three times a day or more [30]. Therefore, toothbrushing twice daily is optimal for caries prevention.

Interdental cleansing tools remove dental plaque in the interdental areas where toothbrushing cannot reach. Interdental toothbrushes, floss, oral irrigators, and tooth cleaning sticks are common self-practice tools for interdental cleaning. A review showed that using an interdental toothbrush or dental floss with toothbrushing reduced more dental plaque than toothbrushing alone [31]. Among interdental cleansing tools, the interdental brush is superior to dental floss. Evidence supporting the use of an oral irrigator or tooth cleaning sticks is not strong [31]. It is noteworthy that the review focused on the effect of plaque control with different interdental cleaning tools. The evidence for interdental cleaning in preventing caries development is limited [31].

Chlorhexidine is a bio-compatible and broad-spectrum antimicrobial disinfectant for plaque control and caries prevention [32]. Various forms of chlorhexidine including varnish, gel, spray, toothpaste, mouth rinse, and chewing gum are available [33]. Common preparations include 0.05% chlorhexidine mouth rinse and 1% chlorhexidine gel. Laboratory studies have demonstrated its effectiveness for plaque control and caries prevention [34]. An analysis of the previous clinical studies revealed that the application of 1% chlorhexidine with 1% thymol varnish on non-cavitated or cavitated root caries showed a 2–3 times greater chance of caries arrest or reversal versus no treatment with low evidence certainty [35]. Clinical studies generally did not support using chlorhexidine for caries control [34]. The evidence for using chlorhexidine gels or varnishes to prevent dental caries or to inhibit *Streptococcus mutans* is weak [33]. No clinical studies on other forms of chlorhexidine for caries prevention have been published. In addition, using chlorhexidine can affect the acidity of the saliva and can increase the cariogenic potential of the oral microbiome [34].

Povidone iodine is another broad-spectrum antimicrobial disinfectant for dental use [36]. Some clinicians use 1% povidone iodine as a pre-procedural mouth rinse. A study showed that it significantly reduces the microorganism concentration in the oral cavity within 4 h after mouth rinsing [37]. Clinicians also applied povidone iodine with fluoride varnish to prevent and to remineralise early caries [38]. A clinical study showed that the combined treatment of povidone iodine and fluoride reduced caries incidence in children with high caries risk [37].

Silver nitrate is a traditional disinfectant for dental use. It is an active ingredient of Howe’s solution for caries management [39]. Because silver is antimicrobial and because fluoride promotes remineralisation, silver nitrate can be used with sodium fluoride to arrest caries [39,40]. A study showed that the combined use of 25% silver nitrate solution and 5% sodium fluoride varnish was effective in arresting early childhood caries [41].

Xylitol is a sugar alcohol that can be added to chewing gum and toothpaste [42]. A clinical trial reported that using fluoride toothpaste containing 10% xylitol reduced caries incidence 13% more than fluoride toothpaste did [42]. However, laboratory studies have suggested that xylitol inhibits the growth of cariogenic bacteria and reduces the cariogenic potential of dental plaque [43,44]. There is insufficient clinical evidence to substantiate using xylitol in caries control [45] and insufficient evidence on the anti-caries effects of other sugar alcohols [42].

Arginine is an amino acid that inhibits the growth of acid-tolerant bacteria [46]. Some toothpaste or mouth rinses contain arginine at different concentrations [47]. Most studies have reported the effect of 1.5% arginine on caries control. A review reported that 1.5% arginine–fluoride toothpaste was superior to fluoride toothpaste and reduced caries incidence by 12% to 21% when compared to the use of fluoride toothpaste [48].

Other antimicrobial components such as chitosan [49], prebiotics [50], and hexetidine [51] have also been used in dental products for plaque control and caries prevention. Their effects have been investigated in clinical studies and have been analysed in systematic reviews. However, evidence to recommend the use of these agents in caries control is insufficient [49,50,51].

### 2.2. Reduction of Caries Risk

A patient’s caries risk can be reduced by identifying caries risk and protective factors, determining the caries risk, and customizing a dental care plan.

Teeth are subjected to demineralisation and remineralisation in the oral cavity. Caries develops when the caries risk factors overwhelm the protective factors [20]. However, caries can be reversed if the protective factors dominate the caries risk factors [52]. Caries risk factors can be catergorised as patient-level and intraoral-level risk factors. Patient-level risk factors such as inadequate oral hygiene practices, inadequate diet, and low recall compliance can be identified by taking a medical and dental history of the patient in question [53]. Intraoral-level caries risk factors, such as the presence of visible plaque and teeth with deep pits and fissures, can be identified through a detailed clinical examination [54]. Caries protective factors can collectively offset the potential pathogenic progress that caries risk factors cause [54]. Protective caries factors include an adequate quantity and quality of saliva, effective oral hygiene practices, appropriate lifestyle or oral habits, sealants, resin infiltrations, etc. [54]. A lack of protective factors can also be viewed as risk factors, which indicate a greater risk of caries.

Caries risk is determined by the total effects of caries risk factors and the protective factors of the patient [54]. Caries risk is the likelihood of the existing lesion progressing or the development of new caries lesions. It is also the prediction of the patient’s caries progression. Because of the complexity of the human disease and the interactions between risk factors and protective factors, it is challenging to evaluate caries risk precisely. To reduce the difficulty and to increase the accuracy of caries risk assessment, several caries risk assessment tools have been developed for dentists for clinical care. Caries management via risk assessment was developed to promote the clinical management philosophy in which the caries disease process is managed according to its etiological factors. It focuses on determining the many factors causing the expression of the disease and takes corrective action [54]. An international caries classification and management system was developed as an evidence-based approach for preventing, reversing, and repairing early caries [55]. It is a set of clinical protocols that address diagnostic, preventive, and restorative decisions for minimal intervention caries treatment. Cariogram was a PC-based software developed to graphically demonstrate a patient’s caries risk [56]. The software program computed and showed caries-risk graphs after inputting the patient’s information. The graphs illustrated the interaction of caries-associated factors and the chances of preventing caries. Besides these assessment tools, the American Dental Association and the American Academy of Pediatric Dentistry offer their caries risk assessment form for dentists to evaluate a patient’s risk of caries development [57].

After determining the caries risk, a personalized dental care plan should be customized for the patient caries control. As a general rule, patients with different caries risks require different dental care plan levels. Dentists can refer to the guidelines of caries risk management systems to customize the dental care plan [54,58].

Toothbrushing with fluoridated toothpaste twice a day, motivational engagement, and dental instructions are recommended for low-risk patients. Dietary intervention, improvement of hyposalivation, sealant or resin infiltration, higher concentration of fluoride products, and more frequent use of fluoride are recommended for patients with moderate and high caries risk [58].

Caries development is related to the intake of free sugar, which refers to all monosaccharides and disaccharides added to foods by the manufacturer, cook, or consumer, plus the sugars that are naturally present in honey, syrups, and fruit juices [59]. Sugar intake guidelines have been developed by WHO to reduce the population’s intake of free sugar [59]. A free sugar intake of less than 10% energy was found to be associated with the reduction of caries incidence in the population [60]. It may be beneficial to limit sugar intake to less than 5% for the minimization of caries risk over one’s life course [60]. Following this guideline, dietary advice for the reduction of caries risk should focus on reducing the amount and the frequency of the consumption of food and drinks containing free sugar. Patients should be encouraged to increase their intake of, all types of fresh fruits and vegetables, nuts, and milk without sugar. This dietary advice should be evidence-based and tailor-made [61].

Fissure sealing is recommended in deep pit and fissure areas or pit and fissure caries for patients with moderate or high caries risk, especially for non-cavitated lesions. A review reported that the application of pit and fissure sealant reduced 11–51% of caries at a follow-up period of up to 48 months [62]. Fissure sealant was found to be effective in arresting occlusal caries in primary teeth at a 44-month follow-up [63]. The success of fissure sealing largely depends on whether the hermetic seal can be achieved [63]. Enough sound dental hard tissue should be assured to provide adequate bonding for an adhesive restoration to seal the caries lesion, especially when there is significant loss of surface integrity in the lesion [64]. Fissure sealant retention is another major concern in terms of its application for caries control. A recent review analysed the retention rate of fissure sealant made from different materials. The results of that review revealed that the polymerizing sealants and resin-modified glass ionomer sealant had a significantly higher retention rate than the conventional glass ionomer sealant [65].

Resin infiltration is indicated for non-cavitated enamel lesions with a lesion depth up to the outer one-third of the dentine in the interproximal surface. The evidence for the sole application of resin infiltration on caries prevention or caries arrest is insufficient. A previous study showed resin infiltration with 5% sodium fluoride varnish had a higher chance of arresting proximal caries compared to no treatment [66]. More clinical studies with a longer follow-up period are required to attest to the effect of resin infiltration on dental caries.

### 2.3. Preservation of Dental Hard Tissue

A dental care plan should be based on the features of sound and carious teeth, the patient’s needs, and available evidence pertaining to dental care [67]. Restorative intervention should mainly be performed to treat cavitated caries lesions that are non-cleansable or lesions that cannot be sealed. It is also indicated in areas where restoration is required to rebuild the aesthetics, structure, or function of a carious tooth [14,68]. According to the FDI task group’s consensus statement, arrested cavitated or non-cavitated caries do not require restorative treatment [67,69]. Active non-cavitated lesions or active cavitate lesions that are cleansable should be treated non-invasively, whereas an active cavitated lesion that is non-cleansable should be restored [67,69]. Non-invasive treatments are recommended if the carious lesion is confined outside of the outer one-third of the dentine. Restorative treatment may be required if a caries extends into the middle one-third of the dentine [67].

Nonrestorative treatment generally aims to remineralise carious enamel and dentine. Topical applications of fluoride or calcium-based products are commonly used to avoid demineralization and/or to promote remineralization. Common fluoride products include sodium fluoride, acidulated phosphate fluoride, sodium monfluorphosphate, stannous fluoride, amine fluoride, and silver diamine fluoride. These fluoride products are delivered in agents such as toothpaste, mouthwash, gel, foams, varnishes, solutions, etc. [70]. The effectiveness of fluoride in controlling dental caries has been extensively investigated (Table 1). Mouth rinse with 200–900 ppm fluoride prevented caries by approximately 23% [71]. Toothpaste with 1000–1500 ppm prevented caries by approximately 25% [72]. Gel with over 10,000 ppm fluoride reduced caries by approximately 28% [73]. Varnish with 22,600 ppm fluoride prevented caries by approximately 43% [74]. Silver diamine fluoride was used to arrest enamel and dentine caries [75,76]. It is a solution with 44,800 ppm fluoride that prevented caries by approximately 78% and arrested 65–91% of caries [77,78].

Common calcium-based products used to remineralise carious lesions are casein phosphopeptide-amorphous calcium phosphate (CPP-ACP), casein phosphopeptide-amorphous calcium fluoride phosphate (CPP-ACFP), and tri-calcium phosphate and nano-hydroxyapatite. Reviews have found evidence that the effect of CPP-ACP and CPP-ACFP on caries control was weak [80]. Therefore, these products are not as effective as fluoride in terms of remineralising caries [80]. Tri-calcium phosphate is used as an additive in some fluoride products, but its bioavailability can be low because it can interact with fluoride [81]. Clinical evidence to support the use of nano-hydroxyapatite for caries management is insufficient.

### 2.4. Long-Term Maintenance

Long-term follow-up and regular recall visits are essential in caries control and for maintaining oral health. The average caries incidence rate is about 0.11 DMFT per person-year [82], while the caries increment is 0.06–0.73 DMFT annually, based on the pooled epidemiological data of 32 cohort studies with a follow-up of at least 3 years [82]. Patients with high caries risk are more likely to have new caries or caries increment even after treatment. Regular recalls are necessary to re-evaluate the therapeutic effects of the caries control strategies that have been employed. Although many dentists have recommended the recall frequency of once every six months [83], the recall interval depends on the patient’s caries risk. The recall interval can be three months for high caries risk patients and two years for low caries risk patients [58]. It is worth noting that the recall interval is often determined based on best practitioner’s clinical judgments since the available evidence on the recall interval is insufficient [83].

## 3. Conclusions

In conclusion, contemporary caries management embraces the philosophy of minimal intervention dentistry, which places the nonrestorative management of dental caries as a priority in all of the stages of caries development. The essential components of nonrestorative management for dental caries include dental plaque control, reducing caries risk, preserving dental hard tissue, and long-term maintenance. Adopting evidence-based strategies for nonrestorative management can prevent, arrest, and/or remineralise dental caries.

## Figures and Tables

**Table 1:** Summary of the meta-analysis of systematic reviews on the effectiveness of common fluoride products in caries prevention.

Topical Fluorides	No. of Studies	No. of Participants	D(M)FS- Prevented Fractions
Fluoride Toothpaste [72]	70	42,300	24% (95%CI 21–28%)
Fluoride Mouthrinses [71]	35	15,305	27% (95%CI 23–30%)
Fluoride Mousse	-	-	
Fluoride Gels [73]	25	8479	28% (95%CI 19–36%)
Fluoride foam	-	-	
Fluoride Varnishes [74]	13	6479	43% (95%CI 30–57%)
Fluoride solutions	-	-	
Silver Diamine Fluoride [79]	2	915	78% (95%CI 68–87%)
